# One-year results of an eHealth intervention on anxiety in patients undergoing abdominal aortic aneurysm surgery: randomized clinical trial

**DOI:** 10.1093/bjsopen/zrae144

**Published:** 2025-01-15

**Authors:** Olga Nilsson, Malin Stenman, Anna Letterstål, Rebecka Hultgren

**Affiliations:** Department of Molecular Medicine and Surgery, Stockholm Aortic Research Group, STAR, Karolinska Institutet, Stockholm, Sweden; Department of Vascular Surgery, Karolinska University Hospital, Stockholm, Sweden; Department of Molecular Medicine and Surgery, Stockholm Aortic Research Group, STAR, Karolinska Institutet, Stockholm, Sweden; Perioperative Medicine and Intensive Care Function, Karolinska University Hospital, Stockholm, Sweden; School of Health, Care and Social Welfare, Mälardalen University, Västerås, Sweden; Department of Molecular Medicine and Surgery, Stockholm Aortic Research Group, STAR, Karolinska Institutet, Stockholm, Sweden; Department of Vascular Surgery, Karolinska University Hospital, Stockholm, Sweden

## Abstract

**Background:**

The longitudinal effects of educational interventions in people with abdominal aortic aneurysm are largely unexplored. This prospective study investigated whether the anxiety-lowering effect of an eHealth intervention observed at the 1-month follow-up is maintained 1 year after abdominal aortic aneurysm surgery.

**Methods:**

Those scheduled for surgical repair of abdominal aortic aneurysm were recruited in a single-centre randomized clinical trial. The control group received care and follow-up per the institution’s standard. The intervention group received an eHealth tool along with psychosocial support. The 1-month results have been reported. The primary outcome measure was the anxiety mean score (HADS-A).

**Results:**

Of 120 included participants, 96 completed the 1-year follow-up (48 in each treatment group). The mean age was 73 years, a majority (86%) were male and 73% were current or previous smokers. Anxiety symptoms measured with the HADS-A decreased over time in both the intervention group (−0.33) and the control group (−0.35, *P* = 0.868). The improvements in anxiety symptoms seen in the short-term follow-up were not sustained at the 1-year follow-up. No significant mean score differences were found in the intention-to-treat or per-protocol analyses.

**Conclusion:**

In this randomized clinical trial, an eHealth intervention did not result in a sustained reduction in anxiety symptoms compared with standard care in the same cohort. The study provides an insight into the limited acceptability of an eHealth tool in people with abdominal aortic aneurysm and valuable data on the recovery trajectory following open and endovascular abdominal aortic aneurysm surgery. Further research is warranted to evaluate the relevance and long-term effectiveness of eHealth interventions in abdominal aortic aneurysm care.

**Registration number:**

NCT03157973 (http://www.clinicaltrials.gov).

## Teaser

In a randomized clinical trial evaluating the effect of an eHealth intervention on anxiety in patients undergoing surgical treatment of abdominal aortic aneurysm, the positive short-term results of the intervention were not sustained at the 1-year follow-up. The study provides data on the natural trajectory of physical and psychological symptoms following abdominal aortic aneurysm repair, and can serve as a basis for future interventions to improve abdominal aortic aneurysm care.

## Introduction

Abdominal aortic aneurysm (AAA) is a fairly common condition in the older population, mainly affecting men and previous smokers^1^. Surgical treatment of AAA is typically offered when the aneurysm reaches a diameter of 5.5 cm or more^2^. Facing surgical treatment of AAA is associated with anxiety; in a previous study the authors found that 20% of patients experienced anxiety symptoms before surgical treatment^3^ whereas another report points to a prevalence of up to 29%^4^. Preoperative anxiety symptoms have been linked to adverse outcomes in patients undergoing surgery, but this has not been evaluated specifically for people with AAA^5,6^. In a systematic review, psychological interventions were found effective in mitigating this surgical stress response^7^.

In recent years, shared decision-making between patients and clinicians has gained increased attention, especially in the context of surgical treatment decisions^8^. However, the trajectory of information during the surveillance interval has been described as uneven with scarce information and with an abundance of information when approaching surgery^9^. Knowledge about the different treatment options and the perioperative process is a requisite for shared decision-making, but patients often have very limited knowledge about the surgical treatment^10,11^. The implementation of decision support tools in the care of AAA has been tested in several studies, but it has been debated that these are tailored to suit the needs of the clinicians rather than meeting the needs of the patients^11,12^. Available online patient information about the surgical treatment has been found to be of low quality^13^. A recent study found that people with AAA warrant earlier written information along with guidance on reliable resources for information^14^.

In spite of irrefutable evidence pointing to the need for improved communication and support during the care trajectory of AAA surgery, no such interventions have been previously evaluated. Guided by a participatory design process, the authors’ research group developed an eHealth intervention to meet the needs of people undergoing surgical treatment of AAA^15^. The intervention comprised a mobile app and psychosocial support by a trained registered nurse. Results from an randomized clinical trial (RCT) evaluating the effect of the eHealth intervention indicated substantial effects on anxiety symptoms at 1 month following surgery^3^. No effects were seen on depression symptoms or health-related quality of life (HRQoL). How these outcomes evolve over time is yet to be explored.

The primary aim of the present study was to evaluate the longitudinal effect of an eHealth intervention on anxiety symptoms in people undergoing surgical treatment of AAA. A secondary aim was to investigate the effect of the intervention on depression symptoms and HRQoL.

## Methods

### Context

The trial was an open, randomized, single-centre trial that ran from November 2016 with a final 1-year follow-up in May 2021. Details of the trial are provided in the primary evaluation of the study^3^. In brief, those planned for elective surgical repair of AAA, age 50 years and above, were considered for the study. Exclusion criteria were diagnosed cognitive dysfunction, severe hearing or visual impairment, inability to speak or understand Swedish, or severe co-morbidity that may hinder the patient from completing the study. After informed consent had been obtained and baseline questionnaires had been completed, participants were randomized to either standard care or intervention. The study was approved by the Regional Ethical Authority (Dnr 2016/1253–31/4), Stockholm, Sweden, and registered in the clinical trials registry (NCT03157973). Written informed consent was obtained from all participants before inclusion in the study.

### Interventions

Details of the intervention have been provided previously^3^, but are summarized below. Study inclusion took place 1 week before scheduled surgery, at which time the participants completed baseline questionnaires, and sociodemographic data along with medical history were collected. Randomization was performed using sealed envelopes in a 1:1 ratio with permuted blocks. The control group obtained oral and written information per the institution’s standards by a vascular surgeon, registered nurse and anaesthetist. The intervention group received the same care, with the addition of an intervention programme consisting of an eHealth tool in the format of a mobile app, and a psychosocial support programme by a contact nurse. The use of the eHealth tool was optional but encouraged, and participants in the intervention group were given a brief introduction to the functionality of the app. Details of the eHealth tool have been described elsewhere^15^. The psychosocial support was performed by a nurse specialist trained in person-centred care and motivational interviewing. Patient preferences guided the content and amplitude of these support sessions, which took place before surgery, at hospital discharge and 3 months after surgery. Participants in the intervention group were also given the possibility to reach out to their contact nurse via telephone as needed. Follow-up questionnaires were scheduled at 1 and 12 months after surgery.

### Instruments

The intervention was evaluated by repeated measures using validated instruments, the Hospital Anxiety and Depression Scale (HADS) and the short-form 12-item health survey (SF-12). HADS aims to measure self-reported symptoms of anxiety and depression in individuals in somatic care^16^. HADS consists of two subscales: HADS-Anxiety (HADS-A) and HADS-Depression (HADS-D), with seven claims for each subscale, and evaluates symptoms during the last 7 days. A cut-off score of eight or more for each subscale has been established as a threshold for mild to moderate anxiety and depression symptoms, such that higher scores indicate more severe symptoms^17^. Thereby, a HADS score greater than or equal to eight on each subscale was registered as anxiety or depression in this study. To explicate the effect of interventions aiming to reduce anxiety symptoms in clinical situations, the minimal clinically important difference (MCID) has been established. The MCID defines the smallest notable change in a treatment outcome that is of clinical significance. For HADS-A, the MCID has been defined as 1.67 when measuring within a treatment group and 1.29 between treatment groups^18^. The SF-12 assesses physical and mental health components and is a reliable and well used instrument^19^. The survey comprises 12 questions regarding the respondents’ social and everyday activities due to physical or mental limitations, restricted to the last week. The SF-12 measures eight dimensions of HRQoL: general health, physical functioning, role limitations (due to physical health), bodily pain, vitality, social functioning, mental health and role limitations (due to emotional health). It produces two components of HRQoL: the physical component summary (PCS) and the mental component summary (MCS). Each component ranges between 0 and 100 and has a mean score of 50 and s.d. of 10 in the general population, with higher PCS or MCS scores indicating a better state of health. Use of the eHealth tool was defined as having logged into the app on one occasion or more. The subset of participants in the intervention group who utilized the eHealth tool are defined as ‘app users’.

### Outcomes

The primary outcome measure was anxiety mean scores measured by HADS-A in an intention-to-treat analysis and in complementary per-protocol analysis of app users in the intervention group *versus* controls. The secondary outcome measures were depression mean scores and HRQoL measured by HADS-D and SF-12 respectively.

### Statistical analysis

Basic descriptive data at baseline was provided. The sample size was estimated for the primary endpoint: HADS-A differences at 1 month after surgery. With an estimated 20% attrition rate, this motivated inclusion of 60 persons in each treatment arm (80% power, 5% significance level)^20,21^. The study arms were compared using the independent sample *t* test for continuous variables with normal distributions. Pearson’s χ^2^ tests were used for discrete variables, Student’s *t* tests for independent samples and Fisher’s exact tests for contingent variables; *P* < 0.050 was considered significant. SF-12 scores were calculated using the Quality Metric Health OutcomesTM PRO CoRE Scoring Software (OptumVR; QualityMetric, Lincoln, RI, USA). Data were analysed using IBM SPSS® statistics v. 27 (IBM, Armonk, NY, USA).

## Results

**Fig. 1:**
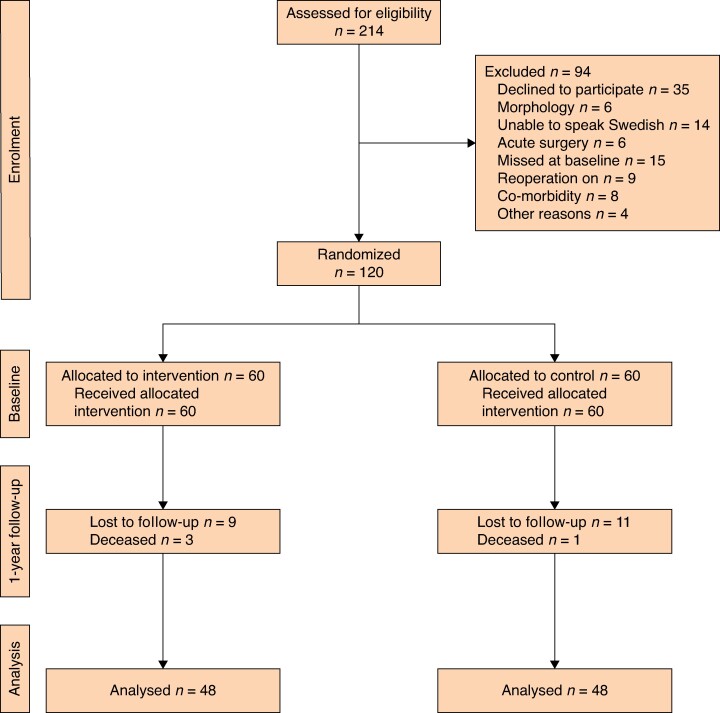
Consort diagram Flow diagram describing the design of the study: enrolment, intervention, follow-up and data analysis.

During the study, 214 persons were approached and invited to participate; of these, 120 were included in the study. In both the intervention group and control group, 48 participants (80%) completed the questionnaires at both baseline and 1 year after surgery and were included in the final analysis (*Fig. 1*). The demographic characteristics of individuals included at baseline have been published previously^3^. Among those who completed the 1-year follow-up, no significant differences in demographic characteristics, co-morbidity, physiological or surgical variables were seen between the treatment groups at baseline (*Table 1*). In the total cohort, the majority (86%) were male, and roughly half underwent OSR (open surgical repair) (52%). The mean age was 73 years. The intervention group had a longer average duration of hospital stay than the control group (8.04 days *versus* 4.95 days, *P* = 0.029).

**Table 1.** Clinical characteristics and surgical outcomes of patients in the control group and intervention group

	Control (*n* = 48)	Intervention (*n* = 48)	*P*
**Demographics**			
Age (years), mean(s.d.)	74(6)	73(7)	0.798
Male	44 (92)	39 (81)	0.232
Never smoker	4 (8)	9 (19)	0.745
**Education level**			1.000*
Junior compulsory	10 (21)	12 (25)	
Senior high school	22 (46)	18 (37.5)	
Postgraduate/university	16 (33)	18 (37.5)	
**Co-morbidity**			
Diabetes mellitus (any)	10 (21)	5 (10)	0.261
Hypertension	35 (73)	39 (81)	0.334
Coronary artery disease	14 (29)	7 (15)	0.084
Cerebrovascular disease	4 (8)	10 (21)	0.083
Peripheral arterial disease	6 (12.5)	5 (10)	0.750
Chronic obstructive pulmonary disease	17 (35)	16 (33)	0.831
Depression	3 (6)	4 (8)	0.203
**Physiologic characteristics**			
Systolic blood pressure (mm Hg), mean(s.d.)	142(16.6)	140(14.4)	0.418
Ankle brachial pressure index	0.93 (0.45)	0.93 (0.49)	0.995
AAA diameter (mm), mean(s.d.)	57.6(5.6)	58.5(5.2)	0.451
**Surgical data**			
EVAR	29 (60)	21 (44)	0.152
Duration of hospital stay (days), mean(s.d.)	4.95(3.56)	8.04(7.89)	0.029
Days in ICU, mean(s.d.)	0.60(0.98)	1.49(2.88)	0.048
Deceased within 30 days	1	0	n/a
**HADS-A at inclusion**			
HADS-A, mean(s.d.)	3.9(4.0)	5.1(3.9)	0.155
HADS-A ≥ 8	8 (16.7)	13 (27.1)	0.324
**HADS-A at 1-year follow-up**			
HADS-A, mean(s.d.)	3.6(4.5)	4.73(5.7)	0.270
HADS-A ≥ 8	9 (19)	13 (27.1)	0.467
**eHealth tool**			
Participants who utilized the tool	n/a	26 (54)	n/a

^*^One-way ANOVA. Values are *n* (%) unless otherwise indicated. AAA, abdominal aortic aneurysm; EVAR, endovascular aortic repair; HADS-A, Hospital Anxiety and Depression Scale-Anxiety; n/a, not applicable.

### Anxiety

**Fig. 2:**
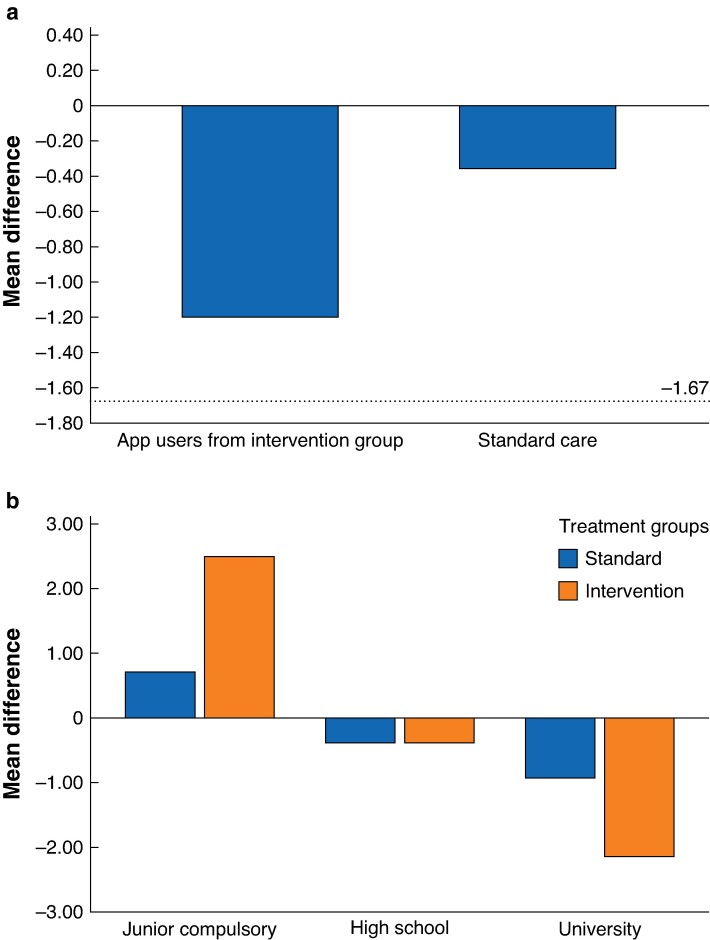
Presentation of mean differences in HADS-A scores from baseline to 1-year follow-up, negative values represent less anxiety The differences are presented for control *versus* app users and educational level split by control *versus* intervention. **a** Differences in HADS-A from baseline to 1 year after surgery, presented by control group *versus* app users in the intervention group. –1.67 is the minimal clinically important difference for HADS-A when measuring within a treatment group. **b** Differences in HADS-A from baseline to 1 year after surgery by educational level, presented by the control group *versus* intervention group. HADS-A, Hospital Anxiety and Depression Scale-Anxiety.

At baseline, there was no significant difference in HADS-A mean scores between the intervention and control groups (5.1 *versus* 3.9 respectively, *P* = 0.155), or in the proportion of participants reporting HADS-A of eight or more (27.1% *versus* 16.7%, *P* = 0.324) (*Table 1*). In the primary analysis of changes in HADS-A from baseline to 1 year, no significant sustained effect on anxiety symptoms could be detected between the intervention and control groups (−0.33 *versus* −0.35 respectively, *P* = 0.868) (*Table 2*, *Fig. 2*). When stratifying for educational level, surgical treatment type and sex, no effect on anxiety mean scores was seen (*Table 2*). The established MCID of 1.67 within groups was reached when stratifying for educational level such that those who had studied at university (*n* = 18) exceeded the MCID effect of the intervention compared with those with a corresponding educational level in the control group (*n* = 16) (−2.167 *versus* −0.937). A reversed association was seen in those with a low educational level, with increased anxiety mean scores at 1 year after surgery in both the control (*n* = 10) and intervention groups (*n* = 12) (0.70 *versus* 2.50) (*Table 2*, *Fig. 2*). Similar results were seen in the per-protocol analysis of users *versus* non-users of the eHealth tool within the intervention group (*Table 2*, *Fig. 2*).

**Table 2:** Changes between baseline and 1-year follow-up scores regarding HADS-A. Subgroup analysis on participants in the intervention group using the eHealth tool. Education level, sex and surgical technique are also presented

	Mean change in HADS-A Baseline to 1 year after surgery		Mean difference between intervention and control (95% c.i.)	*P* *
	Control (*n* = 48)	Intervention (*n* = 48)		
Total cohort	−0.35	−0.33	−0.02 (−1.93,1.89)	0.868
Use of the eHealth tool	Control	App users (*n* = 25)		
	−0.35	−1.19	0.84 (−1.35,3.03)	0.487
**Education level**	Control (*n* = 48)	Intervention (*n* = 48)		
Junior compulsory	0.70	2.50	−1.80 (−5.91,2.31)	0.497
High school	−0.41	−0.39	−0.02 (−3.26,3.22)	0.778
University	−0.94	−2.17	1.23 (−1.80,4.26)	0.597
**Surgical technique**				
OR	−0.16	−0.78	0.62 (−2.51,3.75)	0.522
EVAR	−0.48	0.24	−0.72 (−2.77,1.33)	0.545
**Sex**				
Male	−0.34	−0.05	−0.29 (−2.43,1.85)	0.788
Female	−0.50	−1.55	1.05 (−5.11,7.23)	0.700

^*^Analysed using the Mann–Whitney *U* test. HADS-A, Hospital Anxiety and Depression Scale-Anxiety; EVAR, endovascular aortic repair; OR, open repair.

### Depression and HRQoL

**Fig. 3:**
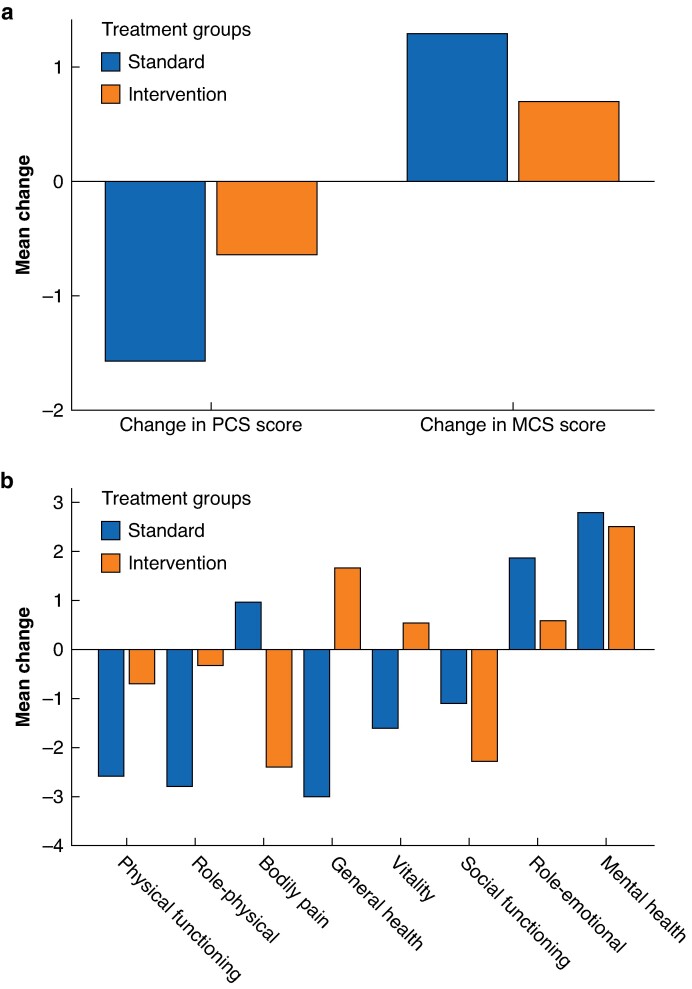
Changes in PCS and MCS mean scores from baseline to the 1-year follow-up by treatment group Changes are presented by PCS and MCS scores and the eight HRQoL dimensions of the SF-12 instrument. **a** Changes in PCS and MCS mean scores from baseline to the 1-year follow-up across treatment groups. **b** Changes in the different dimensions of SF-12 from baseline to the 1-year follow-up across treatment groups. HRQoL, health-related quality of life; PCS, physical component summary; MCS, mental component summary; SF-12, 12-item short form health survey.

In the primary analysis of changes in HADS-D from baseline to 1 year, no significant difference in HADS-D mean scores was detected between the control group and intervention group (*Table 3*, *Fig. 3*). The secondary, per-protocol analysis on the effect of the eHealth tool did not result in significant effects on HADS-D or SF-12. In subgroup analyses of the secondary outcomes stratified by sex and surgical treatment method, no difference was seen (*Table 3*). PCS mean scores (mean(s.d.)) at the 1-year follow-up were 44.9(10.23) and 46.3(10.6) for the control group and intervention group respectively. No significant mean difference in PCS from baseline to follow-up was seen when comparing the control and intervention groups (−3.18 *versus* −1.30, *P* = 0.296). MCS mean scores (mean(s.d.)) were 53.95(10.67) for the control group and 51.20(13.24) for the intervention group; no significant difference could be detected from baseline to follow-up (2.55 *versus* 1.37 for the control group and intervention group respectively, *P* = 0.629). PCS decreased over time for both treatment groups, whereas MCS increased (*Table 3*, *Fig. 3*).

**Table 3:** Changes between scores from baseline to 1 year regarding HADS-D and SF-12 respectively. Subgroup analysis on participants in the intervention group using the eHealth tool

	Mean change		Mean difference between intervention and control (95% c.i.)	*P*
	Control	Intervention		
HADS-D	−0.28	0.50	0.78 (−2.33,0.77)	0.799
**SF-12**				
PCS	−3.18	−1.30	−1.88 (−5.41,1.66)	0.296
MCS	2.55	1.37	1.18 (−3.69,6.06)	0.629
				
	Control	App users (*n* = 25)	Mean difference app users *versus* control group	
**PCS** Use of the eHealth tool	−3.18	−1.60	1.58 (−2.55,5.71)	0.425
**MCS** Use of the eHealth tool	2.55	3.44	0.89 (−4.45,6.24)	0.767

HADS-D, Hospital Anxiety and Depression Scale-Depression; SF-12, 12-item short form health survey; MCS, mental component summary; PCS, physical component summary.

## Discussion

In this RCT, the combined intervention of an eHealth tool and psychosocial support showed a positive effect on anxiety symptoms 30 days after surgery but similar sustained effects could not be detected 1 year after surgical treatment of AAA. Furthermore, no significant effects of the intervention on the secondary outcome measures of depression symptoms and HRQoL were seen. The results are in line with the intention-to-treat analysis at the 1-month follow-up, which could not detect significant effects between the treatment groups^3^. However, the per-protocol analysis revealed that those who utilized the eHealth tool had significantly lower anxiety symptoms after surgery. Nonetheless, the uptake of the intervention was limited and only 50% of those offered the eHealth tool chose to use it^3^. In a qualitative evaluation of the intervention, participants in the intervention group described that they actively postponed the use of the eHealth tool as they were afraid that it would contain information that might propagate their worry^22^. Participants maintained their usual information seeking behaviour, and those who were unfamiliar with mobile technology were unwilling to engage in using the eHealth tool.

In the present study, users of the eHealth tool were younger and had a higher educational level^3^. Low socioeconomic status is a known barrier to the use of eHealth services but this reluctance can be mitigated by iterative design and the validation of intervention content with end-users^23^. Although the eHealth tool was developed in co-design with patients, the timing of the introduction of the eHealth tool 1 week before surgery may have caught the participants at a vulnerable time point, rendering them insusceptible to the intervention. Another important factor to acknowledge when implementing eHealth interventions is the target groups’ acceptance of such an intervention. Although the technical advancements in recent decades offer new opportunities to provide patients with individualized psychoeducational interventions, older patients may not be susceptible to the technology. Determinants of low digital health literacy are increasing age, low educational level, low income and low social support^24^. These results stress that future initiatives to improve the digital literacy of patients in the field of vascular surgery are pivotal to mediate these discrepancies and improve the accessibility to eHealth services, irrespective of age or socioeconomic factors.

Although there are a variety of reports on the association between diagnosis, treatment and a negative influence on quality of life in people with AAA, there is a surprising paucity of interventional trials in order to modulate such associations^25–27^. Since there is a general lack of supportive interventions in the AAA patient group, long-term effects on well being have not been investigated or reported previously. The general short- and mid-term influence of the disease or treatment on peoples’ well being has to some extent been explored, but again without reports of supportive interventions.

The natural course of recovery after AAA surgery differs depending on the surgical treatment method. Previous studies report that people undergoing OSR have decreased physical and mental health up to 3 months after surgery with normalized functioning at 1 year after surgery^28^. The effect of endovascular aortic repair (EVAR) treatment on HRQoL and physical functioning is seemingly less aggravating. EVAR may, however, have more protracted effects and in the long term, open repair seems superior to EVAR in terms of HRQoL and health status^28^. The current results show the slight decrease in physical functioning, measured with the PCS score of the SF-12, seen at the 1-month follow-up persisted at the 1-year follow-up^3^. This is in line with previous studies, where early deterioration in the physical domains have been noted irrespective of the method of surgical treatment^28^.

The gradual decline in physical functioning is of course expected in this cohort and may pertain to advanced age and the elevated cardiovascular morbidity rate, which has been previously described in people with AAA^27^. In a cross-sectional study of men at a mean follow-up time of 37 months following elective AAA surgery, HRQoL was significantly lower in older age groups^29^. The Swedish norm sets for SF-12 assessments in the older population report similar patterns, with a gradual decrease in HRQoL in persons over the age of 75 years^30^. In the present study, the PCS and MCS mean scores for both the control group and intervention group were both slightly higher than the Swedish norm scores for older populations, indicating that 1 year after surgery, their HRQoL is comparable to the general age-matched population^30^.

The HADS has good psychometric properties and has been used extensively in vascular surgery; it has also proven to be valid and useful in screening for symptoms of depression and anxiety in surgical patients^31^. Furthermore, it has been shown to adequately capture psychological distress among a general population of 65–80 year olds^32^. However, a scoping review found that the perspectives of people with AAA do not align with generic quantitative scales^33^. In a qualitative evidence synthesis, the psychological impact of AAA diagnosis and surgery may rather pertain to fear of rupture and lack of control^34^, symptoms not captured by the HADS instrument. Due to the lack of a disease-specific instrument, HADS may underestimate the psychological distress in people with AAA owing to insufficient coverage of assessed symptoms.

Although comparable to the age-matched population, the decline in physical functioning 1 year after an elective AAA repair seen in the present study highlights the need for adequate, balanced preoperative information regarding the postoperative recovery trajectory. This does, in turn, bring a deeper understanding to the reported low relative survival reported for those treated for AAA, and again emphasizes that long-term follow-up of secondary preventive measures of both physical and psychological aspects are needed in order to alter the predestined compromised long-term outcome regardless of surgery in people with AAA^27,35^.

Given the rapid progression in digitalization of healthcare services along with increased digital literacy among the elderly, the case for eHealth interventions is strengthened^36,37^. The trajectory and outcomes of perioperative anxiety are not fully understood in people undergoing AAA surgery. However, in a prospective study of 1771 people undergoing various types of elective surgery, 30% presented with clinically significant anxiety at baseline and those with preoperative anxiety reported higher postoperative opioid use and increased surgical pain^6^. Anxiety levels were normalized at the 6-month follow-up. Although the positive effects of an eHealth intervention on anxiety at 1 month after surgery did not persist at the 1-year follow-up, similar eHealth interventions can and should be considered a viable and useful supplement to standard preoperative care routines. In the present study, participants were recruited irrespective of their baseline HADS-A scoring. While this may have impacted their susceptibility to the intervention, it enables a more thorough description of the natural course of anxiety in this cohort.

The present study is methodologically robust, with a randomized trial design and prospective, longitudinal measurements using validated instruments. Furthermore, the attrition level was acceptable, which strengthens the internal validity of the results^38^. The single-centre design was chosen for logistical reasons, but although clinical routine differs somewhat between vascular centres, the transferability of the findings to other settings is deemed acceptable. The protracted recruitment interval was partly due to the perceived inability of potential participants to utilize mobile applications, a large proportion of potential participants therefore declined to participate. Although a majority had smart phones, few reported using mobile applications to access health information. This further points to the need for initiatives to increase the uptake of eHealth services among groups with lower digital literacy and serves as a reminder to offer alternative information sources according to the patient’s individual needs. Future developments of such services should presumably be developed in co-design with patients and introduced earlier during the surveillance interval.

## Data Availability

The data that support the findings of this study are available from the corresponding author (O.N.) upon reasonable request. The data are not publicly available as they contain information that could compromise the integrity of the participants.
